# Contrasting Isotype-1 β-Tubulin Genotypes at Benzimidazole-Resistance Loci in *Haemonchus contortus* from Yining and Tacheng, Northwestern Xinjiang, China

**DOI:** 10.3390/pathogens15101068

**Published:** 2026-10-08

**Authors:** Waresi Tuersong, Dilare Xuekelaiti, Ailixire Maimaiti, Jianlong Li, Qingyong Guo

**Affiliations:** 1 College of Veterinary Medicine, Xinjiang Agricultural University, Urumqi 830052, China; dilara0118@163.com (D.X.); pbb0108@163.com (J.L.); 2 Xinjiang Key Laboratory of New Drug Research and Development for Herbivorous Animals, Urumqi 830052, China; 3 Xinjiang Regional Key Laboratory of Clinical Veterinary Medicine Research, Urumqi 830052, China; 4 Veterinary Medicine Postdoctoral Research Station, Xinjiang Agricultural University, Urumqi 830052, China; 5 Veterinary Research Institute, Xinjiang Academy of Animal Husbandry, Urumqi 830013, China; 13629926418@163.com

**Keywords:** *Haemonchus contortus*, isotype-1 β-tubulin, E198A, F200Y, SS/rS/rr genotypes, Xinjiang

## Abstract

Benzimidazole (BZ) resistance in *Haemonchus contortus* constrains sheep production worldwide. Northwestern Xinjiang is an important livestock region, yet a parallel comparison of isotype-1 β-tubulin genotypes at the three canonical BZ-resistance loci has not been reported for Yining and Tacheng. Between July and August 2025, 148 adult male worms (77 from Yining and 71 from Tacheng) were collected from sheep processed at local abattoirs. Adult males were identified morphologically by the asymmetrical dorsal ray and the organisation of the bursal rays; females showed a barber-pole appearance. No other abomasal nematode species were observed in the examined material. Species identity of the 148 genotyped males was confirmed by ITS-2 PCR, with Sanger sequencing of 22 amplicons. Remaining males were identified by morphology only and were not subjected to molecular identification. A fragment of the isotype-1 β-tubulin gene spanning codons 167, 198 and 200 was amplified and Sanger-sequenced. Genotypes were coded SS, rS and rr. F167Y was absent from all worms. The two samples differed at E198A and F200Y. The Tacheng sample contained only heterozygous (rS) genotypes at these codons, with substitution-allele frequencies of 11.3% (E198A) and 13.4% (F200Y); SS genotypes predominated. The Yining sample contained rr genotypes at both codons, with higher substitution-allele frequencies (44.8% for E198A and 20.1% for F200Y) and more frequent co-occurrence of non-SS genotypes at the two codons. These findings provide a side-by-side molecular comparison of the two areas. Because sampling was abattoir-based, phenotypic assays were not performed, and individual treatment histories were unavailable, the results should not be read as confirmed clinical resistance or as a basis for region-specific drug protocols. They provide a baseline for further surveillance and for field efficacy testing of benzimidazoles in northern Xinjiang.

## 1. Introduction

*Haemonchus contortus* is a blood-feeding gastrointestinal nematode that parasitizes the abomasum of ruminants and can cause anaemia, emaciation, diarrhoea and even death in the host [1,2]. It is one of the most important pathogens threatening the production of small ruminants such as sheep and goats worldwide [3,4,5]. For decades, benzimidazoles have been the mainstay anthelmintics for the prevention and control of haemonchosis because of their broad spectrum, high efficacy, low cost and ease of administration [6]. However, long-term, repeated and often improper use has driven the widespread emergence of benzimidazole resistance in *H. contortus* globally, seriously threatening the sustainable development of the sheep industry [7].

Benzimidazoles act on parasite β-tubulin: they bind β-tubulin, block microtubule polymerization and disrupt essential processes such as cell division, thereby exerting their anthelmintic effect [8]. Numerous studies have shown that benzimidazole resistance in *H. contortus* is primarily associated with three point mutations (F167Y, E198A and F200Y) in the isotype-1 β-tubulin gene [9]. The frequencies of these mutations vary among geographically distinct isolates. F200Y predominates in many parts of Europe and the Americas, where F167Y is also reported at variable frequencies [8,10]. By contrast, published surveys from China have not detected F167Y, and E198A is often the predominant substitution at these loci [11,12,13,14,15].

Xinjiang is a pivotal sheep-breeding base in China, and haemonchosis is widely reported in its pastoral areas. Molecular surveys of the three β-tubulin loci in local *H. contortus* have expanded in recent years. Zhang et al. sequenced the isotype-1 β-tubulin gene of 183 adult worms from Kezhou and Kashgar in southern Xinjiang and identified E198A as the predominant substitution [13]. Tuerhong et al. (2024) likewise detected only E198A and F200Y, with no F167Y variants, in isolates from Hejing and Minfeng counties of southern Xinjiang [14]. Those earlier reports are cited here for the presence of the substitutions; their classification of heterozygotes is not adopted in the present paper. A subsequent survey by Tuersong et al. (2026) of *H. contortus* from Zhaosu and Tekes counties within Yili Prefecture reported E198A substitution-allele frequencies exceeding 50% at both sites, while F200Y frequencies differed between sites (9.3% in Zhaosu versus 33.3% in Tekes); non-SS genotypes at both codons were also common in the Tekes isolates [15]. Taken together, the available regional datasets show that genotype profiles and allele frequencies of *H. contortus* are not uniform across surveyed parts of Xinjiang.

Existing molecular investigations in Xinjiang have been largely confined to scattered counties in the south and to parts of Yili Prefecture. Comparable data remain limited for Yining City, the capital of Yili Prefecture, and Tacheng City, the administrative centre of Tacheng Prefecture, two important sheep-rearing hubs in northern Xinjiang. Although Xiao et al. (2020) carried out a preliminary survey of *H. contortus* from Tacheng and detected no substitutions at the three loci [16], substitutions at these loci have since been reported from multiple sites in Xinjiang, making an updated comparison of Yining and Tacheng warranted. Previous studies each surveyed a single region in isolation; no parallel comparison of genotype distribution at the three loci between *H. contortus* from Yining and Tacheng has been reported. Yining lies within Yili Prefecture but is geographically separate from the Zhaosu and Tekes counties surveyed previously [15]. The present study therefore extends, rather than duplicates, the earlier work by comparing isolates from these two northern hubs. Individual flock treatment histories were not available for the present collection, and published site-specific records of anthelmintic use in Yining and Tacheng remain scarce. Accordingly, this study was designed as a molecular comparison of abattoir-derived samples rather than as an assessment of local dosing practices.

To address these gaps, we collected adult nematodes from sheep processed at abattoirs in Yining and Tacheng, confirmed the identity of genotyped males by ITS-2 PCR and sequencing, and then amplified, Sanger-sequenced and analysed the three β-tubulin loci (F167Y, E198A and F200Y). Our aims were to describe the distribution of SS, rS and rr genotypes, the substitution-allele frequencies and the genotypic composition of the two samples, and to provide baseline molecular data for continued surveillance of *H. contortus* in northern Xinjiang.

## 2. Materials and Methods

### 2.1. Study Area and Sampling Design

Adult nematodes were collected from sheep slaughtered in Yining City and Tacheng City, northwestern Xinjiang, China. These two cities are major sheep-rearing hubs in northern Xinjiang and have temperate pastoral climates suitable for the development and transmission of gastrointestinal nematodes. Individual anthelmintic treatment histories of the sampled animals were not available. Benzimidazoles are commonly used in sheep production in the region, but flock-specific records of drug class, dose and treatment frequency could not be obtained for the animals examined here. Anthelmintic use was therefore not treated as a measured variable in this study.

Sampling was conducted from July to August 2025. In total, 44 sheep carcasses available at local slaughter facilities were inspected. The geographic origin of each animal was recorded from abattoir registration documents and, when possible, by oral confirmation from the person presenting the animal. Each inspected carcass, and therefore each abomasum, came from a different animal presented by a different person. After slaughter, individual flock identity could not be independently verified. The number of source flocks represented in each abattoir collection is therefore unknown, and flock-level replication was not assumed. The Yining and Tacheng groups refer to geographic samples defined by the abattoir-recorded origin of the host; they should not be interpreted as closed, farm-structured populations.

Natural nematode infection was found in 34 abomasa (77.3% of inspected carcasses), comprising 16 sheep from Yining and 18 sheep from Tacheng. The contents of each abomasum were rinsed and sieved to recover helminths. Adult females were identified by the characteristic barber-pole appearance. Adult males were identified morphologically as *H. contortus* by the asymmetrical dorsal ray and the general organisation of the bursal rays, including the dorsal lobe and subventral rays. No other abomasal nematode species were observed in the examined material.

From each infected abomasum, three to five intact adult males suitable for DNA extraction were taken for genotyping. This yielded 148 worms (77 from Yining and 71 from Tacheng), corresponding to a mean of 4.8 genotyped worms per infected sheep in Yining and 3.9 in Tacheng. Selection was based on specimen integrity and was therefore a convenience sample of recovered males, not a formal random draw from all worms in each abomasum. The 148 males used for genotyping were confirmed as *H. contortus* by ITS-2 PCR (Section 2.3). The remaining males were identified only by morphology and were not subjected to molecular identification.

Each selected worm was washed at least three times in physiological saline to reduce residual host mucus, digesta and intestinal contents; transferred to its own 1.5 mL sterile tube; labelled with sampling locality, a unique serial number and the collection date; and stored at −20 °C until molecular analysis. Genomic DNA was later extracted and genotyped from each worm individually, so each of the 148 sequences represents a single nematode. Individual host identifiers were not retained in the final genotyping dataset; consequently, the exact contribution of each sheep and the mixture of genotypes within a single host could not be tabulated.

### 2.2. Genomic DNA Extraction

Only adult male worms were used for molecular testing, to ensure high DNA purity and to avoid contamination from eggs in the uteri of females. Genomic DNA was extracted individually from each of the 148 adult male specimens (77 from Yining and 71 from Tacheng) using the DNeasy Blood and Tissue Kit (Qiagen, Hilden, Germany) according to the manufacturer’s instructions. These 148 males were the worms subsequently confirmed by ITS-2 PCR and genotyped at the β-tubulin loci (Section 2.3, Section 2.4 and Section 2.5). DNA concentration and purity were assessed on a NanoDrop spectrophotometer (Thermo Fisher Scientific, Waltham, MA, USA). Only samples with an A260/A280 ratio of 1.8–2.0 were retained; samples failing this threshold were re-extracted. Qualified DNA was stored at −20 °C until PCR.

### 2.3. Molecular Identification

To confirm the morphological identification of the 148 males selected for genotyping, the ribosomal second internal transcribed spacer (ITS-2) was amplified by PCR. All 148 genomic DNA samples were amplified using species-specific primers (forward, 5′-CAAATGGCATTTGTCTTTTAG-3′; reverse, 5′-TTAGTTTCTTTTCCTCCGCT-3′) [14].

Each 25 µL reaction contained 2 µL template DNA, 1 µL each of the forward and reverse primers (10 µmol/L), 12.5 µL 2× TransStart FastPfu Fly PCR SuperMix (TransGen Biotech, Beijing, China) and 8.5 µL nuclease-free water. Cycling conditions were: 94 °C for 5 min; 35 cycles of 94 °C for 30 s, 55 °C for 30 s and 72 °C for 30 s; and a final extension at 72 °C for 5 min. A no-template negative control was included in every batch. A previously confirmed *H. contortus* DNA sample was used as a positive control in runs prepared for gel documentation. Products were separated on 1.0% agarose gels stained with ethidium bromide and visualized under UV to confirm a single amplicon of ≈265 bp.

All 148 samples produced a single band of the expected size. Amplicons from 22 worms (11 from Yining and 11 from Tacheng) were then purified and subjected to bidirectional Sanger sequencing (Qingke Biotechnology Co., Ltd., Wuhan, China) to verify that the species-specific PCR products were *H. contortus*. Reads were assembled, aligned and compared against validated reference sequences in the NCBI GenBank database by BLASTn 2.17.0+. The remaining recovered males were not included in this PCR and were identified by morphology only.

### 2.4. Amplification and Sequencing of the Isotype-1 β-Tubulin Gene

A 385 bp fragment of the isotype-1 β-tubulin gene spanning the three codons at benzimidazole-resistance loci (167, 198 and 200) was amplified with the primers reported by Zhang et al. [12]: β-tub-F (5′-GACGCATTCACTTGGAGGAG-3′) and β-tub-R (5′-CATAGGTTGGATTTGTGAGTT-3′). Reactions used the same 25 µL formulation as in Section 2.3, and the cycling programme was identical except for a 55 °C annealing temperature. A no-template negative control was included in every run. Products were checked on 1.0% ethidium–bromide-stained agarose gels; the expected 385 bp fragment was obtained from all 148 worms, purified with a DNA Clean-up Kit (Cowin Biotech, Taizhou, China) and sequenced bidirectionally by Sanger sequencing (Qingke Biotechnology Co., Ltd., Wuhan, China).

### 2.5. Analysis of SNPs at Benzimidazole-Resistance Loci

Sequencing electropherograms were visually curated and genotyped in SnapGene v6.0.2. Sequences were screened for three well-characterized SNPs at benzimidazole-resistance loci: F167Y (TTC→TAC) at codon 167, E198A (GAA→GCA) at codon 198 and F200Y (TTC→TAC) at codon 200. Genotypes were assigned from the chromatogram peak profiles following the criteria of Zhang et al. [12], but were coded according to the convention that the substitution allele is treated as recessive and that heterozygotes are not classified as resistant [17]:

Homozygous susceptible (SS): a single peak corresponding to the wild-type base at the target codon.

Heterozygous (rS): overlapping double peaks at the locus, with the secondary peak exceeding 50% of the primary peak height.

Homozygous (rr): a single peak corresponding to the substituted base at the target codon.

SS, rS and rr are genotype labels only. They do not by themselves constitute phenotypic proof of benzimidazole resistance.

Because direct Sanger sequencing cannot resolve the gametic phase of worms heterozygous at both codon 198 and codon 200, such individuals were recorded as multilocus genotypes rather than as phased haplotypes.

For each geographic sample (Yining, *n* = 77; Tacheng, *n* = 71), genotype frequency (GF) and substitution-allele frequency (SAF) were calculated as:

GF (%) = (number of individuals with the target genotype/total number of worms) × 100;

SAF (%) = (2 × number of rr individuals + number of rS individuals)/(2 × total number of worms) × 100.

## 3. Results

### 3.1. Parasite Collection and Morphological Identification

A total of 2346 adult worms morphologically consistent with *H. contortus* were recovered from the abomasa of 34 naturally infected sheep (16 from Yining and 18 from Tacheng). The overall infection frequency was 77.3% among the 44 carcasses inspected at the abattoirs. Of the recovered worms, 1030 came from Yining and 1316 from Tacheng. Infection intensity varied among hosts, with worm burdens ranging from 51 to 468 per abomasum. These figures describe the inspected carcasses and should not be read as a flock-level prevalence survey.

Adult females showed the characteristic barber-pole appearance. Adult males were identified by the asymmetrical dorsal ray and the general organisation of the bursal rays, including the dorsal lobe and subventral rays (Figure S1). No other abomasal nematode species were observed in the examined material. For genotyping, three to five intact adult males were taken from each infected abomasum, giving 148 individually processed worms (77 from Yining and 71 from Tacheng). These 148 males were confirmed by ITS-2 PCR. The remaining males were identified only by morphology and were not subjected to molecular identification.

### 3.2. Molecular Confirmation of Species Identity

ITS-2 PCR produced a single amplicon of approximately 265 bp from all 148 genotyped adult male specimens (Figure S2). Amplicons from 22 worms (11 from Yining and 11 from Tacheng) were then sequenced bidirectionally to confirm the species-specific PCR. BLASTn comparison against GenBank showed 99% nucleotide identity with validated *H. contortus* reference sequences.

### 3.3. SNPs at Benzimidazole-Resistance Loci in the Isotype-1 β-Tubulin Gene

The target 385 bp fragment of the isotype-1 β-tubulin gene was successfully amplified and sequenced from all 148 worms.

**Codon 167.** No variation was detected at this locus; every worm from both samples carried the homozygous susceptible wild-type genotype (TTC/TTC).

**Codons 198 and 200.** Nucleotide substitutions were identified at codon 198 (E198A, GAA→GCA) and codon 200 (F200Y, TTC→TAC). Three genotypes were distinguished from the chromatograms—homozygous susceptible (SS), heterozygous (rS) and homozygous (rr) (Figure S3). Alignment with the wild-type β-tubulin reference confirmed these two amino-acid substitutions, and no other non-synonymous mutations were detected within the sequenced fragment.

### 3.4. Genotype and Allele Frequencies

No polymorphism was found at codon 167; all worms from both samples were homozygous susceptible (Table 1). The two samples differed in the observed genotype profiles at codons 198 (E198A) and 200 (F200Y).

At codon 198, the Tacheng sample comprised only homozygous susceptible (77.5%, SS) and heterozygous (22.5%, rS) genotypes, with no rr worms. In the Yining sample, SS accounted for 33.8% of isolates, whereas rS and rr reached 42.9% and 23.4%, respectively (Table 1).

At codon 200, rr homozygotes were again absent in Tacheng (73.2% SS, 26.8% rS). In Yining, 22.1% of worms were rS and 9.1% were rr (Table 1).

These percentages are descriptive summaries at the level of individual worms. They are not inferential comparisons between hosts or source flocks.

### 3.5. Multilocus Genotype Composition at Codons 198 and 200

Because direct Sanger sequencing cannot resolve the gametic phase of doubly heterozygous individuals, worms were classified by their combined genotype at codons 198 and 200 (multilocus genotype) rather than by inferred haplotype (Table 2). The two samples differed in composition. Tacheng was dominated by the fully susceptible genotype (SS-198/SS-200), which accounted for 63.4% of individuals; the remaining worms carried only heterozygous combinations—rS-198/SS-200 (9.9%), SS-198/rS-200 (14.0%) and rS-198/rS-200 (12.7%)—and no rr genotype at either codon was detected.

In Yining, the fully susceptible genotype was still present (20.8%). Other combinations were more frequent: rr-198/SS-200 reached 23.4% and SS-198/rr-200 was found in 9.0% of individuals, while rS-198/SS-200 and rS-198/rS-200 occurred at 24.7% and 18.2%, respectively (Table 2). These counts show co-occurrence of non-SS genotypes at the two codons in the Yining sample. They do not resolve haplotype phase and should not be read as evidence of genetic linkage, co-selection, or phenotypic resistance.

### 3.6. Substitution-Allele Frequencies

Substitution-allele frequencies (SAFs) are shown in Table 3. At codon 198 (E198A), the A allele was more frequent in the Yining sample (44.8%) than in the Tacheng sample (11.3%). At codon 200 (F200Y), the Y allele reached 20.1% in Yining and 13.4% in Tacheng.

No inferential test was applied to these frequencies. The values summarize the 148 individually genotyped worms and do not estimate flock-level allele frequencies.

## 4. Discussion

This study provides a parallel molecular comparison of isotype-1 β-tubulin genotypes at benzimidazole-resistance loci in *H. contortus* from Yining City and Tacheng City, two important sheep-rearing hubs in northwestern Xinjiang. Consistent with most molecular surveys from other parts of Xinjiang, the F167Y substitution was absent from all 148 worms, and only E198A and F200Y were detected. The uniform absence of F167Y is consistent with previous Chinese datasets in which this codon is not a primary marker [11,12,13,14,15], in contrast to the F200Y-dominated pattern reported in much of Europe and the Americas [10,18,19,20,21,22,23]. In the present samples, the E198A substitution was the more frequent of the two detected changes, in line with the E198A-dominant pattern described across surveyed Xinjiang pastoral zones [13,14,15].

The two abattoir-derived samples differed in the observed distribution of SS, rS and rr genotypes. Tacheng contained only heterozygous (rS) genotypes at E198A and F200Y, with no rr genotypes, and SS genotypes made up more than 60% of the worms. The substitution-allele frequencies were 11.3% for E198A and 13.4% for F200Y. This profile is broadly consistent with the 2020 survey of Tacheng, in which no substitutions at these loci were detected [16]. The low-frequency heterozygotes recorded here show that these alleles are now present in the Tacheng sample; they do not, by themselves, establish the timing or intensity of any intervening selection.

Homozygous (rr) genotypes were detected only in the Yining sample, at both codon 198 (23.4%) and codon 200 (9.1%), and the E198A substitution-allele frequency was 44.8%. Multilocus genotypes that were rr at one codon, as well as double heterozygotes, were also more frequent in Yining (Table 2). These observations indicate a higher frequency of rr genotypes and of the substitution alleles in the Yining worms than in the Tacheng worms. They should not be interpreted as confirmed phenotypic resistance, as “advanced dual-codon resistance”, or as evidence that substitution alleles are becoming fixed. The E198A allele frequency of 44.8% remains well below fixation, and the study is cross-sectional.

The contrast between samples was larger at E198A than at F200Y. This pattern is compatible with stronger relative enrichment of E198A in the Yining sample, which accords with other Xinjiang datasets in which E198A is the predominant substitution at these loci [13,14]. It does not demonstrate that E198A confers a stronger selective advantage under local drug pressure, because treatment histories and phenotypic assays were not available.

Differences in farm structure, benzimidazole use, drug rotation or animal movement are possible explanations for the contrast between Yining and Tacheng, but they were not measured in this study. Individual flock treatment records could not be obtained, and the number of source farms represented in each abattoir collection is unknown. Any attribution of the genotype difference to husbandry or dosing practice should therefore be treated as a hypothesis.

The multilocus counts are consistent with co-occurrence of E198A and F200Y in individual worms, particularly in Yining. Because direct Sanger sequencing cannot phase doubly heterozygous individuals, the data do not establish genetic linkage, co-selection, or independent evolution of the two substitutions. Haplotype-level analysis would require phase-resolved methods, such as cloning, allele-specific assays or long-read sequencing.

Several limitations follow directly from the sampling design. All worms were recovered from commercial abattoirs, so the results describe the inspected carcasses rather than a designed sample of northern Xinjiang flocks. Three to five intact adult males were genotyped from each infected abomasum, but individual host identifiers were not retained; within-host genotype mixing could not be quantified, and worms from the same animal are not independent observations. In addition, genotyping detects polymorphisms at known loci only. No egg-hatch test, larval-development assay or faecal egg count reduction test was performed, and complete deworming histories of the source flocks were unavailable. Accordingly, the present frequencies cannot be translated into a predicted rate of benzimidazole treatment failure, nor into a recommendation to rotate a specific alternative anthelmintic class.

The practical implication of these data is therefore limited to surveillance. The Yining sample carried a higher frequency of rr and compound non-SS genotypes than the Tacheng sample. That contrast justifies continued molecular monitoring in both areas and field efficacy testing of benzimidazoles before any change in control practice is proposed. The study does not claim that the two substitutions are newly discovered, nor that local records from other Chinese provinces are lacking; it reports a direct comparison of two under-sampled northern hubs.

## 5. Conclusions

This study provides a parallel molecular comparison of isotype-1 β-tubulin genotypes at known benzimidazole-resistance-associated loci in *Haemonchus contortus* recovered from abattoir-derived sheep samples from Yining and Tacheng. F167Y was not detected. Homozygous substitution (rr) genotypes at E198A and F200Y were detected in Yining but not in Tacheng, where SS and rS genotypes were observed at both loci. The substitution-allele frequencies of E198A and F200Y were higher in Yining than in Tacheng. Given the established association of these substitutions with benzimidazole resistance, particularly F200Y, these data provide a molecular baseline for monitoring future changes and potential increases in resistance-associated allele frequencies in both regions. Continued molecular surveillance, together with field-based efficacy testing, will be valuable for informing local parasite control programmes.

## Figures and Tables

**Table 1:** Genotype frequencies of benzimidazole-resistance-associated SNPs at codons 167, 198 and 200 of the isotype-1 β-tubulin gene in *Haemonchus contortus* from Tacheng and Yining.

Region	Codon	Homozygous Susceptible, SS (%)	Heterozygous, rS (%)	Homozygous, rr (%)
Tacheng (*n* = 71)	167	71 (100)	0 (0)	0 (0)
	198	55 (77.5)	16 (22.5)	0 (0)
	200	52 (73.2)	19 (26.8)	0 (0)
Yining (*n* = 77)	167	77 (100)	0 (0)	0 (0)
	198	26 (33.8)	33 (42.9)	18 (23.4)
	200	53 (68.8)	17 (22.1)	7 (9.1)

SS, homozygous susceptible; rS, heterozygous; rr, homozygous for the substitution at the indicated codon.

**Table 2:** Multilocus genotype frequencies (%) at codons 198 and 200 of the isotype-1 β-tubulin gene in *Haemonchus contortus* from Tacheng and Yining.

Region	SS-198/SS-200	rS-198/SS-200	SS-198/rS-200	rS-198/rS-200	SS-198/rr-200	rr-198/SS-200
Tacheng	45 (63.4)	7 (9.9)	10 (14.0)	9 (12.7)	0 (0)	0 (0)
Yining	16 (20.8)	19 (24.7)	3 (3.9)	14 (18.2)	7 (9.0)	18 (23.4)

SS, homozygous susceptible; rS, heterozygous; rr, homozygous for the substitution. Genotypes are given as codon 198/codon 200.

**Table 3:** Substitution-allele frequencies (%) at codons 198 and 200 of the isotype-1 β-tubulin gene of *Haemonchus contortus* from Tacheng and Yining.

Region	Codon 198 (E198A)		Codon 200 (F200Y)	
	Substitution Allele (A)	Wild-Type Allele (E)	Substitution Allele (Y)	Wild-Type Allele (F)
Tacheng	11.3	88.7	13.4	86.6
Yining	44.8	55.2	20.1	79.9

A, alanine (E198A substitution); E, glutamic acid (wild-type); Y, tyrosine (F200Y substitution); F, phenylalanine (wild-type). Allele names do not imply a phenotypic diagnosis of resistance.

## Data Availability

All other data supporting the findings are available from the corresponding authors on reasonable request.

## Supplementary Materials

The following supporting information can be downloaded at: https://www.mdpi.com/article/10.3390/pathogens15101068/s1. Figure S1. Morphological identification of adult *H. contortus*. (A) Adult worms attached to the abomasal wall; (B) Isolated adult nematodes; (C) characteristic twisted body of a female; (D) Y-shaped copulatory spicules of a male. Figure S2. Representative agarose-gel electrophoresis of ITS-2 PCR products from adult male *H. contortus*. Lanes 1–11, individual worms from Yining; lanes 12–22, individual worms from Tacheng; lane 23, positive control (*H. contortus* DNA); lane 24, no-template negative control; lane M, 2000-bp DNA ladder. All samples produced a single band of the expected size (265 bp). Figure S3. Representative sequencing chromatograms of benzimidazole resistance-associated SNPs in the isotype-1 β-tubulin gene of *H. contortus*. (A) Homozygous resistance-associated genotype at codon 198 (GCA, E198A); (B) homozygous resistance-associated genotype at codon 200 (TAC, F200Y); (C) heterozygous genotype at codon 198 (GAA/GCA); (D) heterozygous genotype at codon 200 (TTC/TAC). Polymorphic nucleotides are highlighted in red boxes.
