# Supplementary material for: Contrasting Isotype-1 β-Tubulin Genotypes at Benzimidazole-Resistance Loci in Haemonchus contortus from Yining and Tacheng, Northwestern Xinjiang, China
Source: Pathogens. 2026 Oct 8;15(10):1068. doi: 10.3390/pathogens15101068 (PMC13708239; doi:10.3390/pathogens15101068)
Supplement: Supplementary file 1 [file pathogens-15-01068-s001.zip › pathogens-4549049-supplementary.pdf]

Supplementary Figures

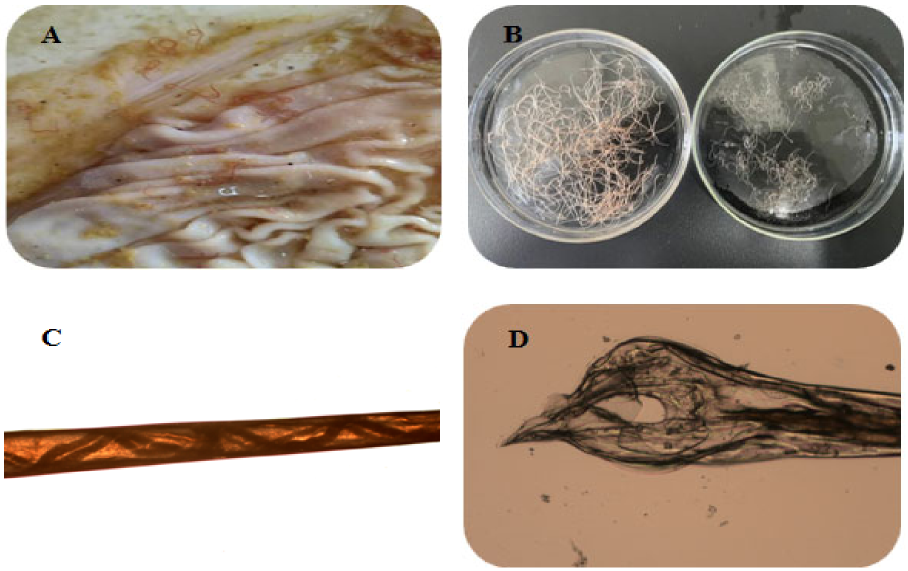

Supplementary Figure S1. Morphological identification of adult *H. contortus*.

(A) Adult worms attached to the abomasal wall; (B) Isolated adult nematodes; (C) characteristic twisted body of a female; (D) Y-shaped copulatory spicules of a male.

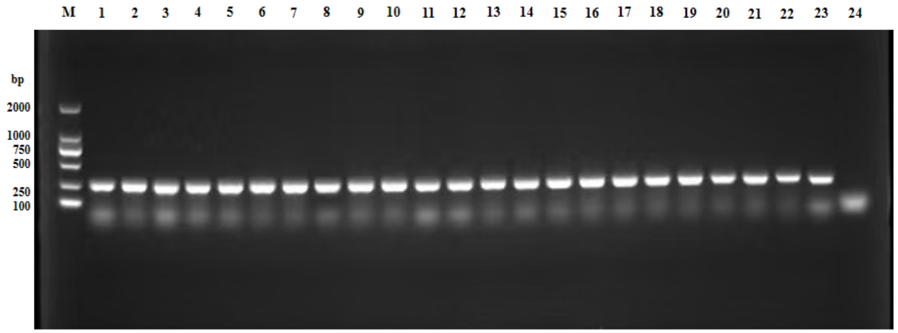

Supplementary Figure S2. Representative agarose-gel electrophoresis of ITS-2 PCR products from adult male *H. contortus*.

Lanes 1–11, individual worms from Yining; lanes 12–22, individual worms from Tacheng; lane 23, positive control (*H. contortus* DNA); lane 24, no-template negative control; lane M, 2000-bp DNA ladder. All samples produced a single band of the expected size (265 bp).

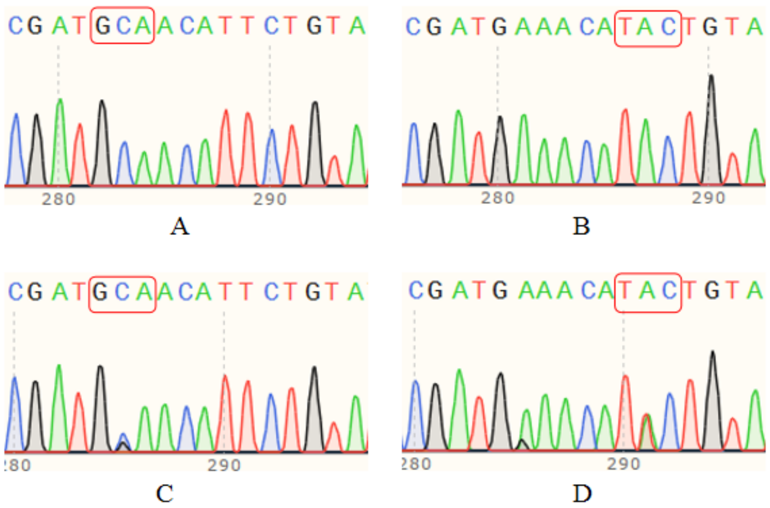

Supplementary Figure S3. Representative sequencing chromatograms of benzimidazole resistance-associated SNPs in the isotype-1 β-tubulin gene of *H. contortus*.

(A) Homozygous resistance-associated genotype at codon 198 (GCA, E198A); (B) homozygous resistance-associated genotype at codon 200 (TAC, F200Y); (C) heterozygous genotype at codon 198 (GAA/GCA); (D) heterozygous genotype at codon 200 (TTC/TAC). Polymorphic nucleotides are highlighted in red boxes.
